# Fast and accurate deformable contour propagation for intra-fraction adaptive magnetic resonance-guided prostate radiotherapy

**DOI:** 10.1016/j.phro.2022.02.008

**Published:** 2022-02-17

**Authors:** Thomas Willigenburg, Cornel Zachiu, Jan J.W. Lagendijk, Jochem R.N. van der Voort van Zyp, Hans C.J. de Boer, Bas W. Raaymakers

**Affiliations:** University Medical Centre Utrecht, Department of Radiation Oncology, 3508 GA Utrecht, The Netherlands

**Keywords:** MRI-guided radiotherapy, MR-guided linear accelerator, Radiotherapy workflow, Intra-fraction adaptation, Prostate cancer

## Abstract

To facilitate full intra-fraction adaptive MR-guided radiotherapy, accurate contour propagation is needed. We aimed to assess the clinical usability of intra-fraction propagated contours by a deformable image registration algorithm in ten prostate cancer patients. Two observers judged the contours on need for manual adaptation and feasibility of adapting contours within 3 min. CTV and bladder contours needed none or only minor editing in most cases (≥ 97%), whereas rectum contours needed more extensive editing in 12–23%. Nevertheless, adaptation times were < 3 min for ≥ 93% of the cases. This paves the way for exploring adaptive workflows using intra-fraction deformable contour propagation.

## Highlights

• Accurate contours are needed for intra-fraction adaption using contour propagation.
• A deformable image registration algorithm (EVolution) was tested for this purpose.
• Clinical quality of intra-fraction propagated contours was assessed by 2 physicians.
• EVolution provided good results in 10 prostate cancer cases.
• This paves the way for adaptive workflows using deformable contour propagation.

## 1 Introduction

External beam radiotherapy treatment is challenged by inter- and intra-fraction anatomical changes in shape, volume, and location of the target and organs-at-risk (OARs) [1], [2], [3], [4]. This can result in a lower dose to the target and/or higher dose to the OARs as compared to the pre-treatment plan [5]. The clinical introduction of magnetic resonance (MR)-guided linear accelerators (MR-Linac) has significantly impacted radiotherapy workflows by enabling MR imaging prior to and during beam-on together with fast planning tools [6], [7], [8], [9], [10]. Currently, MR-Linac systems allow for non-rigid inter-fraction adaptation by daily imaging, re-contouring, and treatment planning [7], [8]. With this approach, the treatment plan is optimized for the daily anatomy just prior to beam-on.

Intra-fractional changes during radiotherapy delivery have become even more important with current interest in extremely-hypofractionated radiotherapy (i.e., ≤ 3 fractions) with larger fractional doses and therefore longer beam-on times [11], [12], [13]. Previously, we have presented intra-fraction motion results in prostate cancer (PCa) [1], [14]. These results demonstrated that to guarantee target coverage with planning target volume (PTV) margins below 5 mm, workflows that allow intra-fraction adaptation are needed. Ultimately, fully automatic online-adaptive workflows may become clinically available, allowing continuous adaptation without operator intervention. Theoretically, the daily adaptive workflow could be repeated multiple times during a single treatment session, delivering the daily fraction in multiple virtual fractions (‘Virtual Fractionation’ [VF]). This would allow accounting for intra-fractional changes. During MR-guided workflows, there is a crucial role for an operator. The operator determines if the propagated contours are acceptable for treatment re-planning and remains responsible [15]. Typically, contours should be manually adjusted after contour propagation before re-planning can be initiated, to obtain representative dose-volume histograms. Current online clinical contour adaptation times in MR-Linac workflows are substantial due to inaccurate propagated contours, with reported inter-fraction contour editing times of over 10 min [15], [16], [17]. Manual contour editing is therefore the major delaying and limiting factor in such a workflow and limits the benefits that theoretically can be obtained.

For workflows using repetitive MR imaging, deformable image registration (DIR), contour propagation, and re-planning to be clinically feasible, a fast and accurate auto-contouring solution is needed that reduces the need for manual adaptation and that limits operator interaction [18]. The aim of this study was to explore the clinical quality of intra-fraction propagated contours produced by a DIR algorithm with respect to need for manual editing and feasibility of editing contours within a short time frame to allow for a fast, online-adaptive workflow for MR-guided PCa radiotherapy.

## 2 Materials and methods

### 2.1 Patients and imaging data

Ten PCa patients treated with 5x7.25 Gy on a 1.5 Tesla MR-Linac (Unity, Elekta AB, Stockholm, Sweden) were included (50 fractions). All patients were part of an institutional review board approved registration and imaging study. During each fraction, an initial daily MR (INI) scan and position verification (PV) scan were acquired (Supplementary Fig. S1). During some fractions, a second PV scan was acquired, i.e., when the time between the PV scan and end of treatment planning was too long due to unforeseen circumstances. In total, 110 MR scans (50 INI, 60 PV) were included (Supplementary Table S1).

### 2.2 Deformable image registration and contour propagation

**Fig. 1:**
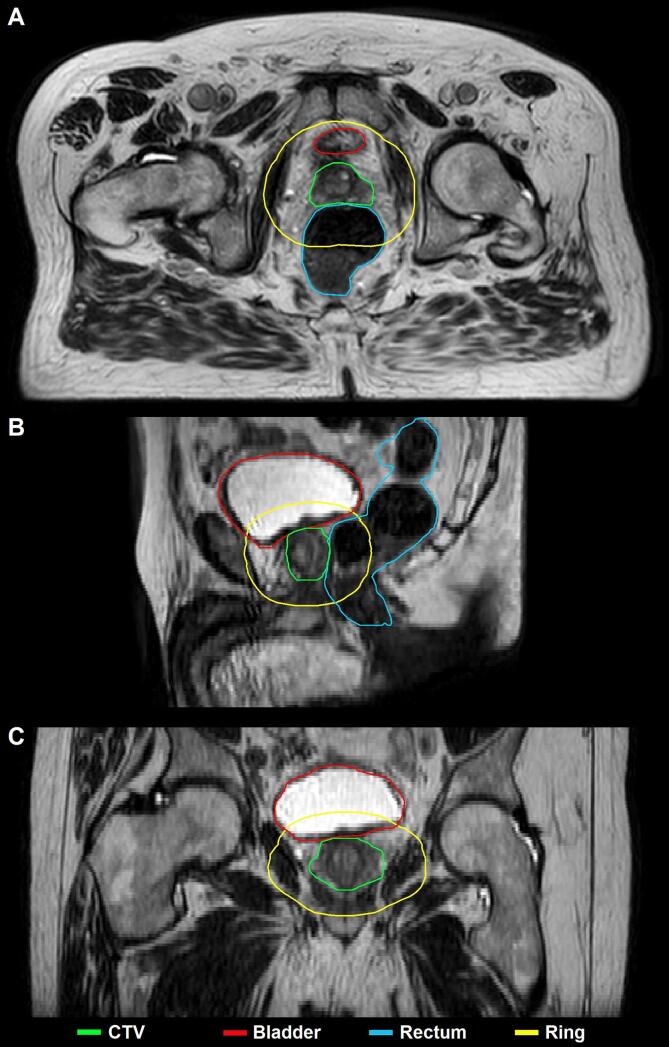
Exemplary propagated deformed contours (solid lines) provided by EVolution on a (A) transversal, (B) sagittal, and (C) coronal slice of the position verification MR scan for a ‘long’ interval case in which no adaptations were necessary within the 2.5 cm ring around the CTV. The CTV is asymmetrical due to the inclusion of the GTV with a 4 mm margin, which is in the left peripheral zone of the prostate. Note some inaccuracies higher up in the deformed rectal contour, outside the ring, due to a large deformation.

For each fraction, the INI scan was registered to the PV scan (n = 50) and the contours were propagated from the INI to PV scan (Supplementary Fig. S2). In case of an additional PV scan (n = 10), the first PV scan was registered to the second PV scan. After registration, the clinical target volume (CTV) and OARs (bladder and rectum) contours were propagated from the prior to the latter scan (Fig. 1). The CTV contour included the prostate body, gross tumour volume (GTV) with a 4 mm margin, and up to 1/3^rd^ of the seminal vesicles.

DIR and contour propagation were performed using the in-house developed EVolution algorithm [19]. The algorithm optimizes the local alignment between similar contrast patterns within the registered images, making it suitable for both mono- and multi-modal image registration. The algorithm was primarily chosen due to its previously demonstrated clinically-acceptable accuracy for contour propagation [18], [20]. Moreover, the method is highly parallelizable, facilitating a fast convergence of < 2 sec for mono-modal MRI registration (256 × 256 × 128 image size) using the Compute Unified Device Architecture (CUDA) and when performed on a NVidia TITAN V graphics processing unit. Finally, it requires a low number of input parameters, which can be maintained at fixed values over the entire duration of a treatment [18], [21]. Therefore, the algorithm can be seamlessly integrated into online adaptive workflows.

### 2.3 Clinical assessment of contours

After DIR and contour propagation, the contours were judged by two independent observers (physicians) on clinical usability using two criteria. First, the need for adaptations within a 2.5 cm ring around the CTV (1.5 cm craniocaudally) was assessed on a four-point scale for each structure separately. The assessment scale ranged from ‘none’ (1) to ‘multiple major adaptations needed’ (4) (Supplementary Fig. S2). The 2.5 cm ring included the region of interest (high-dose region). Second, it was judged if approving and manual editing of all contours could be executed within 3 min, to allow for sufficiently fast cycle times. Results were stratified by observer and the interval between the sequential MR scans (< 10 min versus ≥ 10 min, ‘short’ versus ‘long’) to assess contour quality for shorter and longer MR scan intervals.

## 3 Results

The mean (standard deviation [SD]) time between MR scans was 5.7 (± 1.4) min and 20.0 (± 5.2) min, for ‘short’ (n = 10) and ‘long’ (n = 50) respectively. Stratified by none/few minor adaptations (score 1–2) or multiple minor/ major (score 3–4) adaptations needed, agreement between observer 1 and observer 2 was 98% (59/60), 95% (57/60), and 85% (51/60) for CTV, bladder, and rectum contour, respectively (Table 1). For ‘long’ interval, it was estimated that 46/50 (92%) and 47/50 (94%) cases (observer 1 and 2, respectively) could be edited within 3 min. Both observers estimated that this would be possible for all (100%) ‘short’ interval cases. The remaining ‘long’ interval cases showed larger intra-fraction rectal deformations, in addition to a need for (minor) adjustment of the CTV and/or bladder contour.

**Table 1:** Need for adaptations of propagated contours as scored per observer, stratified by ‘Short’ and ‘Long’ interval between MRI scans.

Adaptions needed		Number of fractions (%)					
		CTV		Bladder		Rectum	
		*Obs 1*	*Obs 2*	*Obs 1*	*Obs 2*	*Obs 1*	*Obs 2*
Short interval (n = 10)							
*None (1)*		8 (80)	8 (80)	10 (100)	9 (90)	4 (40)	8 (80)
*Few minor (2)*		2 (20)	2 (20)	0 (0)	1 (10)	5 (50)	1 (10)
*Multiple minor/few major (3)*		0 (0)	0 (0)	0 (0)	0 (0)	1 (10)	1 (10)
*Multiple major (4)*		0 (0)	0 (0)	0 (0)	0 (0)	0 (0)	0 (0)
Long interval (n = 50)							
*None (1)*		28 (56)	41 (82)	30 (60)	39 (78)	13 (26)	26 (52)
*Few minor (2)*		21 (42)	9 (18)	18 (34)	9 (18)	24 (48)	18 (36)
*Multiple minor/few major (3)*		1 (2)	0 (0)	1 (3)	2 (4)	8 (16)	4 (8)
*Multiple major (4)*		0 (0)	0 (0)	1 (3)	0 (0)	5 (10)	2 (4)

Legend: CTV = clinical target volume; Obs = observer. The corresponding scores (1-4) as provided by the observers (see Fig. S2) are presented within brackets.

## 4 Discussion

We have explored and demonstrated the clinical usability of intra-fraction propagated contours provided by a DIR algorithm for MR-guided PCa radiotherapy treatment. Contours should be generated in a quick and accurate manner, to minimize operator interaction and to maximize the potential benefits adaptive workflows can offer when delivering large fractional doses. Our results suggest that intra-fraction contours provided by EVolution were in general directly acceptable (CTV and bladder) or mostly needed only minor manual editing (rectum). Although manual adaptation was needed in some cases, it could probably be performed within 3 min in the far majority of the fractions.

Online adaptive radiotherapy workflows come with specific needs in terms of DIR technology. Algorithms need to be fast, accurate, and easy to use for the operator. While there are many registration algorithms available in the literature, very few fulfil these requirements, and even fewer have been validated for clinical use. For this work, we selected EVolution based on its demonstrated accurate performance for MR-to-MR contour propagation [18], [20]. The results obtained in the current study are in good correspondence with previous reports, since EVolution delivered overall clinically usable propagated contours. This was particularly the case for instances in which the time interval between sequential MR scans was shorter. In these cases, gradual volume changes and translations, due to bladder filling or drifts of the prostate [1] were less extreme. Our results thus suggest that short cycle times (times between two MR images) are an important factor in the clinical accuracy of intra-fraction propagated contours, and they should therefore be kept as short as possible. The main source of inaccuracies stemmed from major deformations occurring within the rectum, for example in case of a large gas pocket. In such instances, we hypothesize that the large magnitude of the deformations together with the significantly different image features introduced by the gas pocket itself has led to the algorithm converging towards a local minimum and in turn causing a local misregistration. Our previous work on intra-fraction motion indicated that these rectal deformations are unpredictable and non-gradual [1], [14], [22], [23]. Especially cases with large rectal deformations could benefit from an adaptive workflow and therefore warrant extra time to assure contours are accurate.

In terms of computational time, the algorithm converged in approximately 1.5–2.0 sec, which ensures smooth progress of online adaptive workflows that are as of now already time consuming (approximately 45 min per fraction for PCa [1]). Furthermore, the algorithm’s control parameters were maintained at fixed values for all registered MR pairs. Once the algorithm has been configured for registering MR images acquired using a particular acquisition sequence, the same configuration can be maintained for any number of registered image pairs [19]. This is beneficial for online adaptive workflows on an MR-Linac, since there is no requirement for online tuning of algorithm parameters. Therefore, EVolution generally fulfils the technical and functional requirements for clinical use in a VF workflow.

This paper is inherently limited by the exploratory design. We did not carry out a full comparison of i.e., different DIR algorithms or other auto-contouring solutions. Our aim was to assess the clinical quality of the contours provided by EVolution, so that it can serve as a basis for our future work regarding intra-fraction adaptive workflows, and not to identify the most accurate auto-contouring solution. We only presented results for mono-modal MR-MR registration, since the intended use is for an MR-only MR-Linac workflow. As presented previously, this generally leads to better results in terms of Dice’s similarity coefficient compared to CT-MR or multi-model MR-MR registration [18]. The results are therefore not applicable to multi-modal image registration. Additionally, only subjective assessments of the contours were conducted. Nevertheless, agreement rates were high for CTV and bladder contours, which mostly needed no or only minor editing (Table 1). We believe that the manual editing of propagated contours – which inherently is a subjective visual judgement by the operator – is the limiting factor. Keeping that in mind, we decided to work from this perspective. Furthermore, the 3 min cut-off for manual editing was arbitrarily chosen, as this cut-off will depend on multiple aspects that have yet to be investigated for the implementation of a VF workflow. This includes primarily the amount of intra-fraction motion that is expected in the time from the end of image acquisition to actual start of beam-on, during which DIR and contour editing are performed. The timings of such a workflow will ultimately affect the final dose distribution and therefore influence the potential benefits. Additionally, clinical goals such as applying 1 mm CTV-PTV margins will guide the process to determine what is needed from a technical point-of-view. Ideally, the time dedicated to visual inspection and manual contour editing is a few seconds, implying that the contours are always spot-on. Until we can fully rely on accurate auto-contouring solutions, operator intervention will remain essential. Finally, the cut-off was set as a benchmark in the light of current manual adaptation times [15], [24].

Besides exploring clinical usability of propagated contours, the clinical feasibility of employing adaptive workflows for MR-guided PCa radiotherapy should be tested. Our current work has focused on the image registration and contour propagation in a standalone pipeline. Future work should include an assessment of technical feasibility when incorporated in a (pre-)clinical VF workflow and certification of workflow software for intended use.

Concluding, the employed DIR algorithm performed well for intra-fraction propagation of bladder and prostate CTV contours. Generally, rectum contours were acceptable, but sometimes needed more manual editing to fit the anatomy. Nevertheless, adaptation times were below 3 min for most cases. This work paves the way for exploring adaptive workflows using intra-fraction DIR, contour propagation, and re-planning.

## Declaration of Competing Interest

The authors declare that they have no known competing financial interests or personal relationships that could have appeared to influence the work reported in this paper.
