# Supplementary material for: Fast and accurate deformable contour propagation for intra-fraction adaptive magnetic resonance-guided prostate radiotherapy
Source: Phys Imaging Radiat Oncol. 2022 Feb 17;21:62–5. doi: 10.1016/j.phro.2022.02.008 (PMC8861825; doi:10.1016/j.phro.2022.02.008)

SUPPLEMENTARY MATERIAL

'Fast and accurate deformable contour propagation for intra-fraction adaptive magnetic resonance-guided prostate radiotherapy'

Table S1 *MRI parameters*

**Parameter**	**Size 1**	**Size 2**
Sequence	T2-weighted 3D	
Field-of-view	400x400x180 mm	400x447x180 mm
Acquisition voxel size	1.5x1.5x2.0 mm	
Reconstructed voxel size	0.78x0.78x2.0 mm	
Repetition time	1635 ms	
Echo time	120 ms	
Flip angle	90 degrees with constant refocusing control of 100 degrees	
Acquisition time	3 min 05 sec	3 min 24 sec

Figure S1 *MR-Linac Adapt-to-Shape workflow for prostate cancer treatment (adapted from Willigenburg et al.* [11]*). CT=Computed Tomography. MR=Magnetic Resonance (Image). ATS=Adapt-to-Shape.*

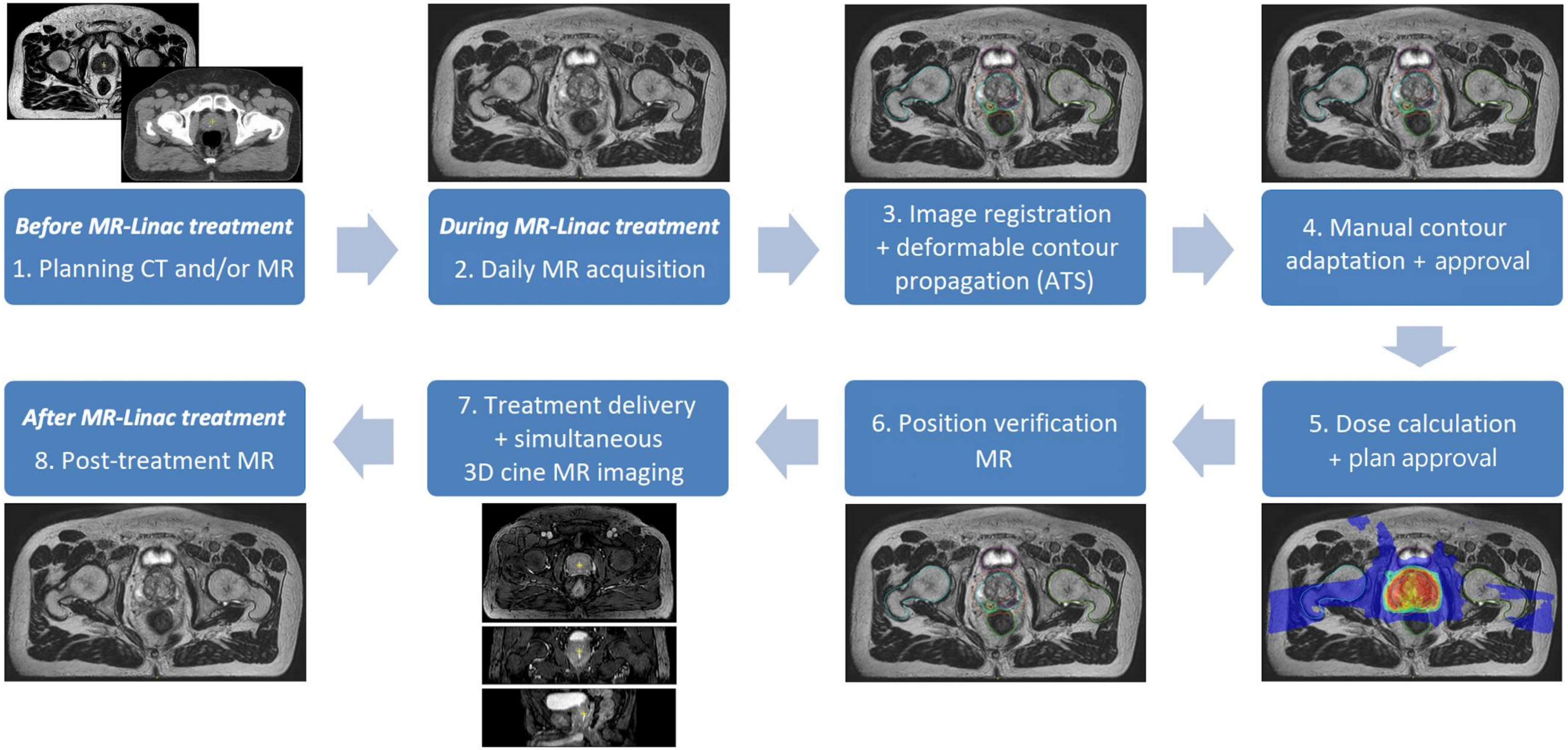

Figure S2 *Flowchart of deformable image registration process and subsequent visual check of contours. MRI=Magnetic Resonance Image. DIR=Deformable Image Registration. CTV=Clinical Target Volume.*

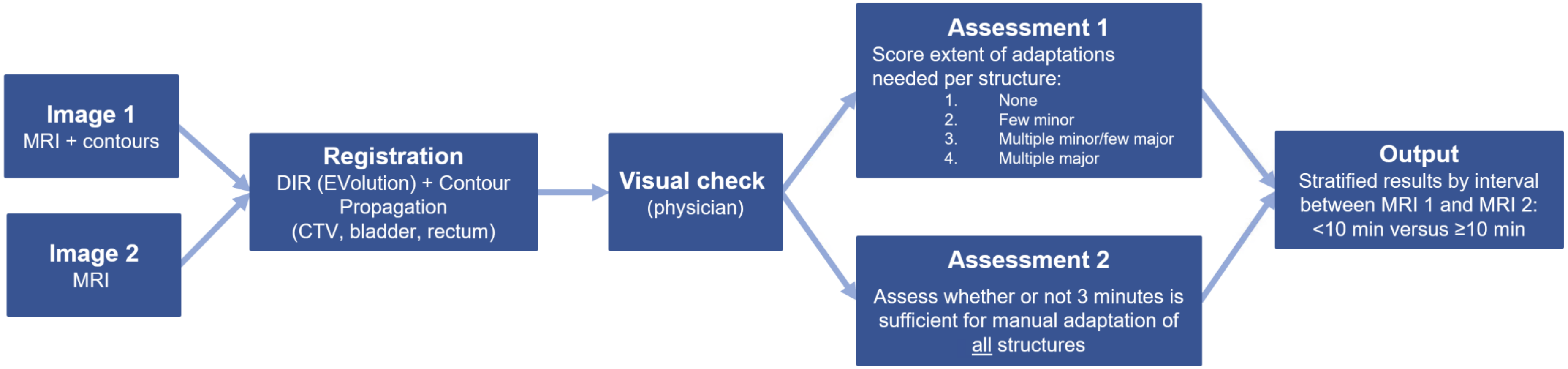

Supplement: Supplementary data 1 [file mmc1.pdf]
